# Cell_motility: a cross-platform, open source application for the study of cell motion paths

**DOI:** 10.1186/1471-2105-7-289

**Published:** 2006-06-08

**Authors:** Lennart Martens, Geert Monsieur, Christophe Ampe, Kris Gevaert, Joël Vandekerckhove

**Affiliations:** 1 Department of Medical Protein Research, Flanders Interuniversity Institute for Biotechnology, Department of Biochemistry, Ghent University, A. Baertsoenkaai 3, B-9000 Ghent, Belgium; 2 The Leuven Institute for Research on Information Systems (LIRIS), Faculty of Economics and Applied Economics, Katholieke Universiteit Leuven, Naamsestraat 69, B-3000 Leuven, Belgium

## Abstract

**Background:**

Migration is an important aspect of cellular behaviour and is therefore widely studied in cell biology. Numerous components are known to participate in this process in a highly dynamic manner. In order to obtain a better insight in cell migration, mutants or drugs are used and their motive phenotype is then linked with the disturbing factors. One of the typical approaches to study motion paths of individual cells relies on fitting mean square displacements to a persistent random walk function. Since the numerous calculations involved often rely on diverse commercial software packages, the analysis can be expensive, labour-intensive and error-prone work. Additionally, due to the nature of algorithms employed the calculations involved are not readily reproducible without access to the exact software package(s) used.

**Results:**

We here present the cell_motility software, an open source Java application under the GNU-GPL license that provides a clear and concise analysis workbench for large amounts of cell motion data. Apart from performing the necessary calculations, the software also visualizes the original motion paths as well as the results of the calculations to help the user interpret the data. The application features an intuitive graphical user interface as well as full user and developer documentation and both source and binary files can be freely downloaded from the project website at  .

**Conclusion:**

In providing a free, open source software solution for the automated processing of cell motion data, we aim to achieve two important goals: labs can greatly simplify their data analysis pipeline as switching between different computational software packages becomes obsolete (thus reducing the chances for human error during data manipulation and transfer) and secondly, to provide scientists in the field with a freely available common platform to perform their analyses, enabling more efficient data quality control through peer reviewing.

## Background

Triggered and directed cell motion is a highly interesting research topic since it is involved in both essential physiological and important pathological processes. Indeed, organism development, tissue repair, inflammation, angiogenesis and tumor metastasis all rely on mobile cells. Correspondingly, the scientific literature abounds with overviews of the importance of cell motility [1-5]. Typically, studies on cell motion can be performed on groups of cells (population assays) as well as on individual cells. As the former depends on the net sum of the motions of the latter, the detailed study of individual cell trajectories can usually reveal greater insights into cell motion behaviour. Additionally, mathematical models have been developed to relate a summation of individual cell paths to population movements [6].

A single cell moving through an isotropic environment will follow an almost straight path over short time intervals, yet exhibit Brownian motion over long time intervals. Overall, this cell motion can be characterized as a persistent random walk [7,8]. The mathematical model to represent this behaviour was convergently deduced from different assumptions by several authors [8,9] and takes the following form for a two-dimensional walk:

<(Δx)^2^> = 2S^2^P [(Δt) - P(1 - e^(-(Δt)/P))]     (eq. 1)

where <(Δx)^2^> is the mean square displacement of the cell over time interval (Δt), S represents the root-mean-square cell speed and P is the persistence time. '^' denotes an exponent.

For long time intervals (ie. t >> P), this formula can be reduced to:

<(Δx)^2^> = 2S^2^Pt     (eq. 2)

If we now take:

D=12S2P     (eq. 3)
 MathType@MTEF@5@5@+=feaafiart1ev1aaatCvAUfKttLearuWrP9MDH5MBPbIqV92AaeXatLxBI9gBaebbnrfifHhDYfgasaacH8akY=wiFfYdH8Gipec8Eeeu0xXdbba9frFj0=OqFfea0dXdd9vqai=hGuQ8kuc9pgc9s8qqaq=dirpe0xb9q8qiLsFr0=vr0=vr0dc8meaabaqaciaacaGaaeqabaqabeGadaaakeaacqWGebarcqGH9aqpdaWcaaqaaiabigdaXaqaaiabikdaYaaacqWGtbWudaahaaWcbeqaaiabikdaYaaakiabdcfaqjaaxMaacaWLjaWaaeWaaeaacqqGLbqzcqqGXbqCcqGGUaGlcqqGGaaicqaIZaWmaiaawIcacaGLPaaaaaa@3C5C@

We can rewrite equation 2 as a standard diffusion equation, with D the diffusion coefficient:

<(Δx)^2^> = 4Dt     (eq. 4)

In order to calculate these mean squared displacements for an individual cell researches typically use a microscope in combination with specialized software to track a single cell over a given time period. This dedicated software ultimately reports a list of X and Y pixel coordinates for the targeted cell, each of which corresponds to the location of the cell at a multiple of a preset time interval. These coordinates are then transformed into standard length measurements (most commonly *μm*), which are subsequently used to calculate mean squared displacements (symbolized by <(Δx)^2^>) as a function of time interval (Δt).

These calculations can be performed in two distinct ways, according to the selection of the user. The first way uses overlapping intervals, calculated according to the following formula [10]:

<(Δx)2>=1(N−n+1)∑i=0N−n[(x(n+i)Δt−xiΔt)2+(y(n+i)Δt−yiΔt)2]     (eq. 5)
 MathType@MTEF@5@5@+=feaafiart1ev1aaatCvAUfKttLearuWrP9MDH5MBPbIqV92AaeXatLxBI9gBaebbnrfifHhDYfgasaacH8akY=wiFfYdH8Gipec8Eeeu0xXdbba9frFj0=OqFfea0dXdd9vqai=hGuQ8kuc9pgc9s8qqaq=dirpe0xb9q8qiLsFr0=vr0=vr0dc8meaabaqaciaacaGaaeqabaqabeGadaaakeaacqGH8aapcqGGOaakcqqHuoarcqWG4baEcqGGPaqkdaahaaWcbeqaaiabikdaYaaakiabg6da+iabg2da9maalaaabaGaeGymaedabaGaeiikaGIaemOta4KaeyOeI0IaemOBa4Maey4kaSIaeGymaeJaeiykaKcaamaaqahabaWaamWaaeaadaqadaqaaiabdIha4naaBaaaleaacqGGOaakcqWGUbGBcqGHRaWkcqWGPbqAcqGGPaqkcqqHuoarcqWG0baDaeqaaOGaeyOeI0IaemiEaG3aaSbaaSqaaiabdMgaPjabfs5aejabdsha0bqabaaakiaawIcacaGLPaaadaahaaWcbeqaaiabikdaYaaakiabgUcaRmaabmaabaGaemyEaK3aaSbaaSqaaiabcIcaOiabd6gaUjabgUcaRiabdMgaPjabcMcaPiabfs5aejabdsha0bqabaGccqGHsislcqWG5bqEdaWgaaWcbaGaemyAaKMaeuiLdqKaemiDaqhabeaaaOGaayjkaiaawMcaamaaCaaaleqabaGaeGOmaidaaaGccaGLBbGaayzxaaaaleaacqWGPbqAcqGH9aqpcqaIWaamaeaacqWGobGtcqGHsislcqWGUbGBa0GaeyyeIuoakiaaxMaacaWLjaWaaeWaaeaacqqGLbqzcqqGXbqCcqGGUaGlcqqGGaaicqaI1aqnaiaawIcacaGLPaaaaaa@783B@

The second method uses non-overlapping intervals, resulting from the following formula [11]:

<(Δx)2>=nN−n∑i=0N−nn−1[(x((1+i)n+1)Δt−x(in+1)Δt)2+(y((1+i)n+1)Δt−y(in+1)Δt)2]     (eq. 6)
 MathType@MTEF@5@5@+=feaafiart1ev1aaatCvAUfKttLearuWrP9MDH5MBPbIqV92AaeXatLxBI9gBaebbnrfifHhDYfgasaacH8akY=wiFfYdH8Gipec8Eeeu0xXdbba9frFj0=OqFfea0dXdd9vqai=hGuQ8kuc9pgc9s8qqaq=dirpe0xb9q8qiLsFr0=vr0=vr0dc8meaabaqaciaacaGaaeqabaqabeGadaaakeaacqGH8aapcqGGOaakcqqHuoarcqWG4baEcqGGPaqkdaahaaWcbeqaaiabikdaYaaakiabg6da+iabg2da9maalaaabaGaemOBa4gabaGaemOta4KaeyOeI0IaemOBa4gaamaaqahabaWaamWaaeaadaqadaqaaiabdIha4naaBaaaleaacqGGOaakcqGGOaakcqaIXaqmcqGHRaWkcqWGPbqAcqGGPaqkcqWGUbGBcqGHRaWkcqaIXaqmcqGGPaqkcqqHuoarcqWG0baDaeqaaOGaeyOeI0IaemiEaG3aaSbaaSqaaiabcIcaOiabdMgaPjabd6gaUjabgUcaRiabigdaXiabcMcaPiabfs5aejabdsha0bqabaaakiaawIcacaGLPaaadaahaaWcbeqaaiabikdaYaaakiabgUcaRmaabmaabaGaemyEaK3aaSbaaSqaaiabcIcaOiabcIcaOiabigdaXiabgUcaRiabdMgaPjabcMcaPiabd6gaUjabgUcaRiabigdaXiabcMcaPiabfs5aejabdsha0bqabaGccqGHsislcqWG5bqEdaWgaaWcbaGaeiikaGIaemyAaKMaemOBa4Maey4kaSIaeGymaeJaeiykaKIaeuiLdqKaemiDaqhabeaaaOGaayjkaiaawMcaamaaCaaaleqabaGaeGOmaidaaaGccaGLBbGaayzxaaaaleaacqWGPbqAcqGH9aqpcqaIWaamaeaadaWcaaqaaiabd6eaojabgkHiTiabd6gaUbqaaiabd6gaUbaacqGHsislcqaIXaqma0GaeyyeIuoakiaaxMaacaWLjaWaaeWaaeaacqqGLbqzcqqGXbqCcqGGUaGlcqqGGaaicqaI2aGnaiaawIcacaGLPaaaaaa@8B3A@

where N represents the total number of steps recorded and n is the step size for the mean squared displacement currently calculated.

The calculations required to transform initial X and Y coordinates into mean squared displacements are already numerous and complex enough to require the assistance of spreadsheets or other specialized software. And even with the help of spreadsheets, there is a very real chance of introducing human error at this early stage.

The second operation, fitting the mean square displacements to the persistent random walk equation, poses a quite different challenge. Due to the nature of the problem, special curve-fitting or non-linear regression algorithms need to be applied which attain a solution through many iterations until a preset convergence is reached. As the solutions generated by these algorithms can be influenced by their specific nature as well as through several user-provided *a priori *parameters (including the maximum number of iterations and the convergence criterion applied), it is often difficult for reviewers or peers to validate results submitted or reported in the literature. On top of this, most researchers apply either proprietary, home-grown algorithms to the task, or use commercial software programs. In either case, reproducing the results requires access to these specific implementations which is often expensive or impossible.

We here present a cross-platform, open source software program to automate the analysis of single cell motions. Our software drastically simplifies the analysis pipeline and eliminates the problem of human error in the calculations. Additionally, since the software is freely available and the full source code can be examined, the calculations can be validated by all interested parties via this common platform. Finally, as the source files can be downloaded, other researchers in the field can extend the software at will to suit their needs.

## Implementation

Cell_motility is an open-source, 100% pure Java application. It can be run on any platform that supports a Java Virtual Machine version 1.4.2 or above. The program offers an intuitive graphical user interface with a cell motion path display, a curve-fitting display and a full report in either plain text, comma-separated values or HTML format for inclusion in word processors, spreadsheets or databases and internet pages.

### 1. Data loading

The cell motion data can be loaded from simple text files, having two columns which can be separated by spaces, tabs, colons or semicolons and which may have a single header line. The user simply needs to point the program to a folder containing one or more of these coordinate text files.

Apart from specifying the source folder, the input dialog allows the user to provide additional details for the processing, such as the time interval used for recording the X and Y coordinates and a length unit. There are four pre-defined units of length measurement (nm, μm, mm and cm) which may be extended by editing a text file bundled with the program. Optionally, the user can also specify a conversion factor to transform the pixel-based coordinates reported by the tracking software into the selected length unit.

A final selection that the user needs to make concerns the desired method for calculating the mean squared displacements (MSD). There are two possible algorithms provided with the software (see 'Background' section above), but it is also straightforward to implement a custom method for calculating MSD and to automatically have it integrated in the software (see section 5 below).

### 2. Calculations

Upon submission of the load parameters, the software starts loading all the data files in the source folder. As soon as a data file is loaded, the mean squared displacements as well as the curve fitting calculations are performed. The user is informed of these proceedings through a progress bar.

The curve-fitting software relies on the Java implementation of the Nelder-Mead Simplex algorithm [12] as freely provided by Dr. Michael Thomas Flanagan [13]. This algorithm will attempt to find the best possible fit for both S and P in equation 1 using an iterative approach. The convergence criterion for these calculations is set to 1e^-6^. The maximum number of iterations allowed when convergence is not reached can be user-defined (see section 4 below) and defaults to 300,000. Typical examples where convergence is not readily reached are highly circular paths and highly persistent paths. Usually, the algorithm will actually be oscillating between two very similar and equally good fits so simple manual verification of these results by means of the curve plot or the goodness of fit should inform the user of the validity of the fit. The results of the calculations as well as the goodness of fit of the persistent random walk equation is visualized as well as reported (see section 3).

### 3. Visualizations and reports

**Figure 1:**
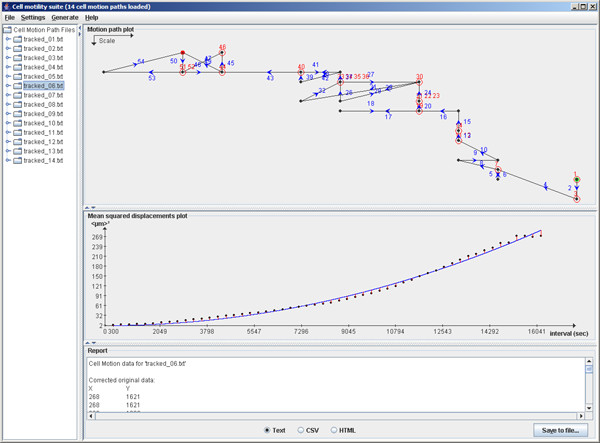
**Screenshot of the cell_motility application**. This screenshot of the application shows the tree-view of the data files on the left, and the three analysis panels on the right. These are (from top to bottom): motion path plot, curve fitting chart and text report.

After loading the data and performing the calculations, the user is confronted with a tree-view of the data files on the left-hand side. Clicking any of these files results in two distinct visualizations and one report for that particular dataset on the right-hand side, a screenshot of this configuration is shown in figure 1. Each of the panels can be resized in both horizontal and vertical direction for optimal viewing.

The top plot shows the actual cell motion path as seen by the microscope. The steps are numbered and a blue arrow shows directionality. Each data point is represented by a black dot except for the start and end location which are shown as slightly oversized green and red dots, respectively. Whenever a cell appeared stationary between two tracking events, that location will be encircled in red and the relevant step number(s) are shown in red as well. On the upper left, a scale is shown with the relative lengths of a single unit in both X and Y direction. The user can shrink or expand the motion plot panel to achieve evenly-spaced X and Y directions if desired

The lower plot shows the mean squared displacements as black dots, the fitted persistent random walk equation in blue and the red lines indicate the distances used to calculate the least sum of squares. This plot allows the user to inspect both the goodness of fit and the nature of the curve – this can be either exponential or linear and provides information about the absence or presence of directionality in the cell motion.

The bottom panel presents a text report of the analysis. This report includes the original data file (albeit with the pixel-to-length-unit conversion, if applicable), the calculated mean squared displacements and the results of the exponential curve fitting. The latter first shows the number of iterations that were required to achieve convergence as well as a note on whether convergence was reached ('complete') or not ('incomplete'). Then the fitted values of S and P are shown. It is noteworthy that S can be a negative value as S is only present in equation 1 in quadratic form and the fitting is therefore invariant to sign opposition. Rather than only displaying the absolute value of S, we decided that reporting of the exact fitted value of S whenever it was fitted as a negative number gives more insight into the workings of the iterative algorithm. Finally, a goodness of fit is reported. This is the sum of square orthogonal distances reported by the fitting algorithm. Ideally, this value should equal 0 (perfect fit), in practice however, this value will be higher. The value is presented to provide objective feedback on the fitting itself as well as to allow objective comparison of the fitting performance against other algorithms.

The bottom panel also sports three radio buttons which allow formatting of the report either as plain text, comma-separated values or HTML. Additionally, the 'Save to file' button enables the user to export the report in its current formatting to a file.

It is also important to note that a full report over all loaded data files can be instantly generated via the 'Generate → Full report' menu of the application.

Another report that is available through the 'Generate' menu is the 'statistical report'. This report details some simple descriptive statistics (the mean and standard deviation) for both S and P, based on all currently loaded cell motion paths.

### 4. User customization

Several parameters concerning the graphical user interface and the curve fitting can be user-defined via the 'Settings' menu of the application. These include the look and feel of the application, the drawing detail of the fitted function and the maximum number of iterations allowed during curve fitting when convergence is not reached. On slower computers, it is often beneficial to lower both the drawing detail of the fitted function as well as the maximum allowed number of iterations. Experience learns that, on the datasets submitted, if convergence is not reached after several tens of thousands of iterations, the fitting will not noticeably improve by allowing even more (up to several million) iterations.

### 5. Extending the software

The software is built around a fully descriptive and frameworked class loading system for the discovery and usage of implementations that can calculate MSD. The developer need only implement a simple interface (*AverageDisplacements*) and specify a label as well as the fully qualified classname in a text file (*AverageDisplacementImplementations.properties*) which should be located in the Virtual Machine classpath. The cell_motility software will then automatically locate and load this new implementation and will display it as an additional choice on the user interface, directly allowing the user to select it.

## Results and discussion

The cell_motility software allows the user to process and analyze the data obtained through cell motion path studies in two dimensions. The software reads text files containing cell coordinates that are recorded over evenly spaced time intervals. From these raw position data, the software reconstructs a graphical view of the original motion path and attempts to fit a random walk model to the MSD of the cells. Computation of the latter can be performed using both overlapping and non-overlapping intervals and it is even straightforward to add custom algorithms to the software without requiring a recompilation of the source code.

The MSD data is then fitted to a persistent random walk model using a Nelder-Mead Simplex non-linear regression algorithm that is both fast and reliable. On average, 100 cell motion paths can be loaded and computed in this way in about one minute using a simple laptop. The resulting fitted curve is displayed in a chart together with the MSD data points. The original data as well as the intermediate results are also reported alongside the end results in the users' choice of plain text, comma-separated value or HTML formatted text. It is furthermore possible to apply a standard statistical analysis on a complete dataset to quickly analyze trends in a population.

The cell_motility software is freely available and completely open source, presenting users with all the necessary details of its functioning as well as allowing researchers detailed control over the actual behaviour if this is desired. Built around frameworks for the processing of data, the software is written to make contributions from other developers in the field easy and efficient, thereby substantially lowering the threshold to community participation. Future features will include the analysis and visualization of three-dimensional cell motion paths as well as an extension of the available data processing algorithms.

## Conclusion

We have developed a simple and easy-to-use software application to automate single-cell motion studies, an important aspect of cell biology. By providing this application as cross-platform, freeware binaries, we aim to provide researchers worldwide with an inexpensive, automated analysis pipeline while simultaneously providing the field with a common platform to validate and reproduce submitted or published findings.

Since the software is completely open source and built around a simple, abstracted framework, it is both easy and convenient for other researchers to extend it with their own algorithms or adaptations and even their own visualizations. The collaborative aspect of third-party contributions is guaranteed through the use of the GNU-GPL license for this software. Additionally, the software resides in a clearly versioned CVS repository to which interested developers can submit their updates.

For the next version of cell_motility we plan to extend the calculations and vizualisations of the software to include three-dimensional tracking of individual cells as well as an implementation of generalized nonlinear least-squares regression for the curve fitting.

## Availability and requirements

Project name

Cell_motility

Project homepage



Operating system(s)

Platform independent

Programming language

Java

Other requirements

Java 1.4.2 or higher

License

GNU GPL

Any restrictions to use by non-academics

None.

## Abbreviations

- eq. equation

- MSD mean squared displacements

## Authors' contributions

LM designed and wrote the cell_motility software and drafted the manuscript.

GM provided the integration with the curve-fitting library.

CA assisted in thoroughly testing the software and helped to finalize the manuscript.

KG coordinated the software development and helped to draft the manuscript.

JV provided project coordination and critically revised the draft manuscript.
